# Chalcones: Synthetic Chemistry Follows Where Nature Leads

**DOI:** 10.3390/biom11081203

**Published:** 2021-08-13

**Authors:** Hiba A. Jasim, Lutfun Nahar, Mohammad A. Jasim, Sharon A. Moore, Kenneth J. Ritchie, Satyajit D. Sarker

**Affiliations:** 1 Centre for Natural Products Discovery (CNPD), School of Pharmacy and Biomolecular Sciences, Liverpool John Moores University, James Parsons Building, Byrom Street, Liverpool L3 3AF, UK; h.a.jasim@uoanbar.edu.iq (H.A.J.); S.Sarker@ljmu.ac.uk (S.D.S.); 2 Department of Biology, College of Education for Pure Sciences, University of Anbar, Al-Anbar 10081, Iraq; 3 Laboratory of Growth Regulators, Institute of Experimental Botany ASCR & Palacký University, Šlechtitelů 27, 78371 Olomouc, Czech Republic; 4 Department of Biology, College of Education for Women, University of Anbar, Al-Anbar 10081, Iraq; mohammed.a.jasim@uoanbar.edu.iq; 5 Faculty of Science and Engineering, University of Wolverhampton, Wolverhampton WV1 1LY, UK; sharonmoore1@hotmail.com

**Keywords:** chalcones, biomolecular interactions, synthesis, natural products, bioactivities, mechanisms, anticancer, antimicrobial, phenolics

## Abstract

Chalcones belong to the flavonoid class of phenolic compounds. They form one of the largest groups of bioactive natural products. The potential anticancer, anti-inflammatory, antimicrobial, antioxidant, and antiparasitic properties of naturally occurring chalcones, and their unique chemical structural features inspired the synthesis of numerous chalcone derivatives. In fact, structural features of chalcones are easy to construct from simple aromatic compounds, and it is convenient to perform structural modifications to generate functionalized chalcone derivatives. Many of these synthetic analogs were shown to possess similar bioactivities as their natural counterparts, but often with an enhanced potency and reduced toxicity. This review article aims to demonstrate how bioinspired synthesis of chalcone derivatives can potentially introduce a new chemical space for exploitation for new drug discovery, justifying the title of this article. However, the focus remains on critical appraisal of synthesized chalcones and their derivatives for their bioactivities, linking to their interactions at the biomolecular level where appropriate, and revealing their possible mechanisms of action.

## 1. Introduction

**Figure 1:**
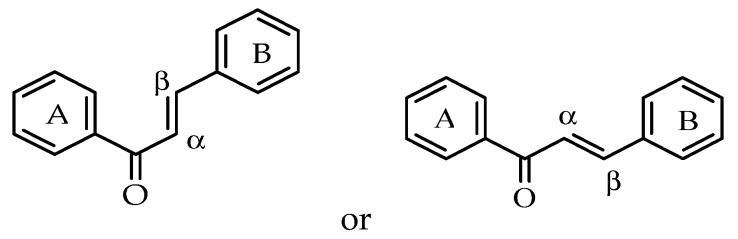
General structure of chalcones.

Chalcones are flavonoid-type phenolic phytochemicals, often referred to as ‘open-chain flavonoids’, and biosynthesized via the shikimate pathway [1]. Chalcones are considered as the biosynthetic precursors of flavonoids. Chemically, chalcones are generally α,β-unsaturated ketones consisting of two aromatic rings (rings A and B) linked through a three-carbon alkenone unit (Figure 1), but these may also include some saturated ketones, commonly known as dihydrochalcones, where instead of a three-carbon alkenone unit, a three-carbon alkanone unit is present. Among the naturally occurring chalcones, the presence of one or more phenolic hydroxyl functionalities is ubiquitous, and prenyl and geranyl substitutions on the aromatic rings are also widely observed. There are several thousand naturally occurring chalcones reported in the literature [2], and many of those chalcones were shown to interact with various biomolecules and possess cytoprotective and modulatory properties, making them potentially suitable candidates for therapeutic interventions in many human ailments. Several patents are also available for chalcones and their derivatives for their activities as anticancer, anti-inflammatory, antimitotic, antioxidant, and cytotoxic properties [3].

Because of structural simplicity and therapeutic potential, several bioinspired syntheses of chalcone derivatives and evaluation of their bioactivities were reported in the literature [2]. The first few attempts for the synthesis of chalcones began in the 1800s and continued through the subsequent centuries [4]. In this review article, some of these synthesized chalcones and their derivatives were critically appraised for their bioactivities, linking to their interactions at the biomolecular level where appropriate, and revealing their possible mechanisms of action. Moreover, this review article fundamentally aims to demonstrate how bioinspired synthesis of chalcone derivatives can potentially introduce a new chemical space for exploitation for new drug discovery, justifying the title of this article. 

## 2. Chalcone Synthesis

**Scheme 1:**
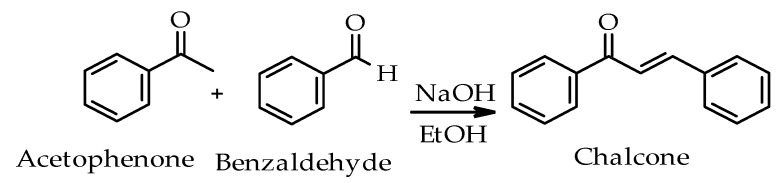
Synthesis of chalcone.

Chalcones are considered privileged structures in the medicinal chemistry due to the relative ease with which they may be produced and modified, reflecting the similar ease with which they are produced in nature. From as early as the 19th century, many researchers have developed synthetic chalcones, with Kostanecki and Tambor being acknowledged as the first to successfully prepare synthetic chalcones using a method involving treatment of *o*-acetoxychalcone dibromides with alcoholic alkali [5,6]. However, the current methods of chalcone synthesis utilize an alkaline base and a polar solvent to couple two compounds with an aromatic ring each, e.g., acetophenone and benzaldehyde, and to produce the core chalcone nucleus (Scheme 1) [7,8,9]. Various aromatic compounds and different methods used in the synthesis of several chalcone derivatives (**2**–**10**) and chalcone itself (**1**) are summarized in Table 1.

## 3. Molecular Mechanisms of Chalcone Actions

Various bioactivities of chalcones result from their interactions with several biomolecules and different signaling pathways. Some of those molecular mechanisms of activities of chalcones are discussed in the following subsections.

### 3.1. Activation of Nuclear Factor-Erythroid 2 p45 Subunit-Related Factor 2 Pathway and Cellular Defence Genes

**Figure 2:**
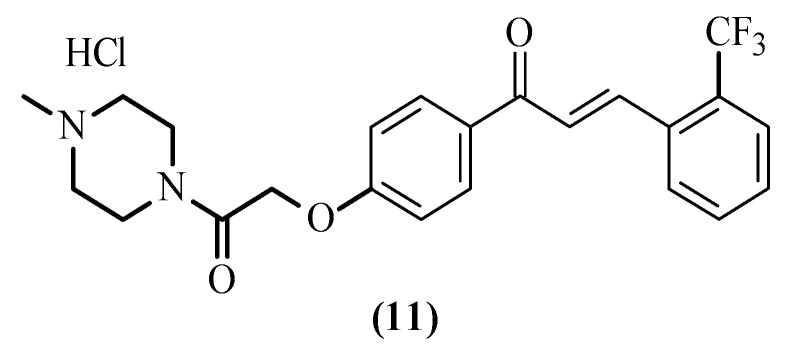
A synthetic chalcone derivative as an Nrf2 activator.

Nuclear factor-erythroid (NF-E2) p45-related factor 2 (Nrf2) is a transcription factor, which controls the expression of a battery of antioxidant genes, all of which have an antioxidant response element (ARE) in their promoter which allows the binding of Nrf2 [10]. The ARE-Nrf2 complex leads to induction of cell expression of cell defense genes. such as glutathione S-transferase (GST), hemoxygenase-1 (HO-1), and xenobiotic metabolizing enzyme (NAD(P)H: quinone oxidoreductase 1) (NQO1) [11,12,13]. Kim et al. [14] synthesized a series of chalcone derivatives, and among them, a new chalcone derivative (**11**) (Figure 2) was found to possess the best Nrf2 activation properties and could induce Nrf2 nuclear translocation. This chalcone salt (**11**) was shown to induce gene expression of antioxidant enzymes and attenuate inflammatory responses in activated microglia. There has been an increasing number of publications that detail the relationship between chalcones and Nrf2, with several studies reporting the ability of chalcones to induce Nrf2. The ability of synthetic and natural chalcones to activate the Nrf2 signaling pathway was reviewed recently [15].

**Figure 3:**
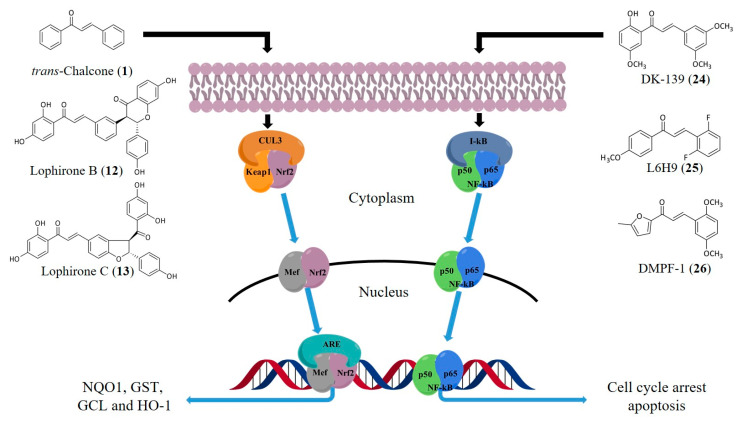
Chalcones that induce transcription factors: chalcone (**1**), lophirone B (**12**) and C (**13**) induce the Nrf2 pathway, while synthetic chalcones DK-139 (**24**), L6H9 (**25**) and DMPF-1 (**26**) induce the NF-κB pathway.

Ajiboye et al. [16] reported that natural chalcones lophirones B (**12**) and C (**13**) could induce Nrf2 (Figure 3). In addition, the natural chalcone, 3,4,2′,4′-tetrahydroxychalcone 4′-*O*-β-D-glucopyranoside (**14**), was noted to increase the level of NQO1 [NAD(P) H: quinone oxidoreductase 1] in Hepa 1c1c7 cells [17]. Martinez et al. [18] reported that chalcone (**1**) could increase glutathione (GSH), HO-1, and Nrf2 (Figure 3). Furthermore, other researchers found 2-chloro-4′,6′-dimethoxy-2′-hydroxychalcone (**15**), a synthetic chalcone, to increase GSH levels by enhancing its biosynthesis [19]. The ability to induce activation of Nrf2 was exploited to assess potential cancer chemopreventive activity of licorice samples collected from different geographical locations [20]. In fact, the structural diversity that exists among synthetic and natural chalcones, ease of structural modification, and the presence of α,β-unsaturated carbonyl functionality (pharmacophore) make these compounds suitable drug candidates or can lead to targeting of Nrf2-dependent disease.

### 3.2. Activation of the Nuclear Factor-Kappa B (NF-κB) PI3K/Akt Signaling Pathway

The transcription factor, nuclear factor-kappa B PI3K/Akt (NF-κB), is a heterodimer consisting of p50 and p65 subunits, and is central to the functioning of the immune system. It has a role to play in the expression of immunoglobulin-k. NF-κB is activated by extracellular promoters, such as growth factors and inflammatory cytokines. The extracellular promoters switch on NF-κB signaling through dissolution and phosphorylation of the inhibiting I-κB protein. Allowing NF-κB to enter the nucleus and regulate cell cycle and apoptosis-related gene expression [21]. 

**Figure 4:**
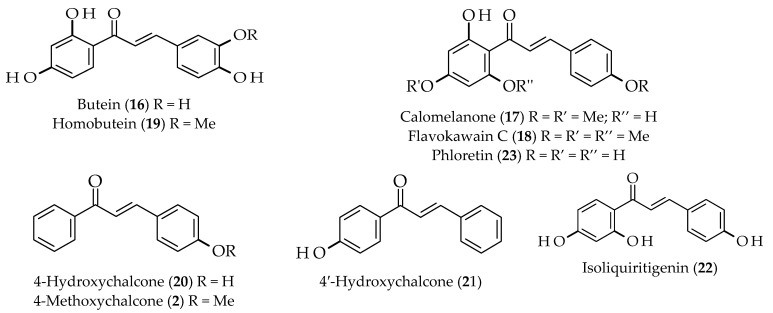
Examples of some naturally occurring chalcones that induce the NF-κB pathway.

Several studies have examined the effect of chalcones on NF-κB signaling as a plausible way to modulate inflammation and cancer, and found that several natural chalcones, e.g., butein (**16**), calomelanone (**17**), flavokawain C (**18**), homobutein (**19**), 4-hydroxychalcone (**20**), 4′-hydroxychalcone (**21**), isoliquiritigenin (**22**), 4-methoxychalcone (**2**), and phloretin (**23**) (Figure 4) have the ability to suppress NF-κB signaling [22]. Similarly, NF-κB activation could be stimulated by several synthetic chalcones, such as 2-hydroxy-3′,5,5′-trimethoxychalcone (DK-139) (**24**) [23], (*E*)-2,6-difluoro-4′-methoxychalcone (L6H9) (**25**) [24], and 3-(2,5-dimethoxyphenyl)-1-(5-methylfuran-2-yl)prop-2-en-1-one (DMPF-1) (**26**) [25] (Figure 3).

### 3.3. Effects on the Cell Cycle

Many researchers have investigated the effects of synthetic and natural chalcones on the cell cycle [26,27,28,29,30]. Dimethyl-cardamonin (**27**) was shown to arrest cell cycle in the G_1_ phase by decreased expression of cyclin D1, CDK4 (cyclin-dependent kinase 4), and phospho-Rb [30], while Hseu et al. [29] reported that the chalcone flavokawain B (**28**) could cause cell cycle arrest in the G_2_/M phase in human oral carcinoma cells.

**Figure 5:**
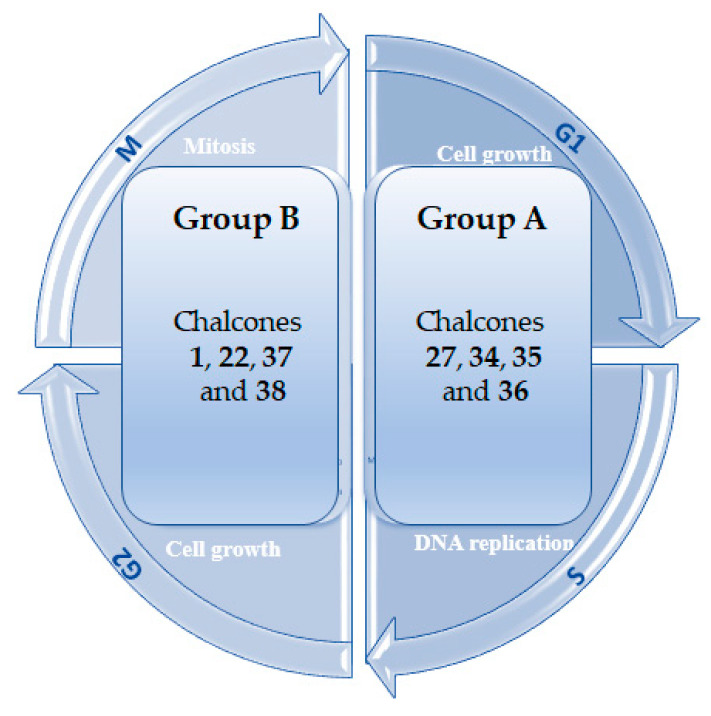
The effect of natural and synthetic chalcones on the cell cycle. Group A: Chalcones arrest the cell cycle at the G_1_/S phase, i.e., dimethyl cardamonin (**27**) [30], tetramethoxychalcone (**34**) [28], 2′-hydroxy-2,3,4′,6′-tetramethoxychalcone (**35**) [34], and tetrahydro-[1,2,4]triazolo[3,4-a]isoquinoline chalcones (**36**) [35]. Group B: Chalcones that arrest the cell cycle at the G_2_/M phase chalcone (**1**) [29], isoliquiritigenin (**22**) [36], (2*E*)-3-(acridin-9-yl)-1-(2,6-dimethoxyphenyl)prop-2-en-1-one (**37**) [37], and quinazolinone-chalcone (**38**) [38].

**Figure 6:**
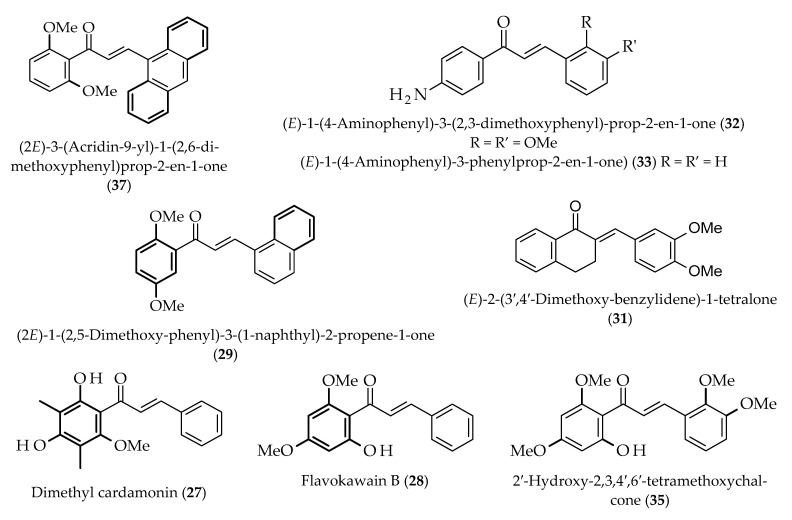

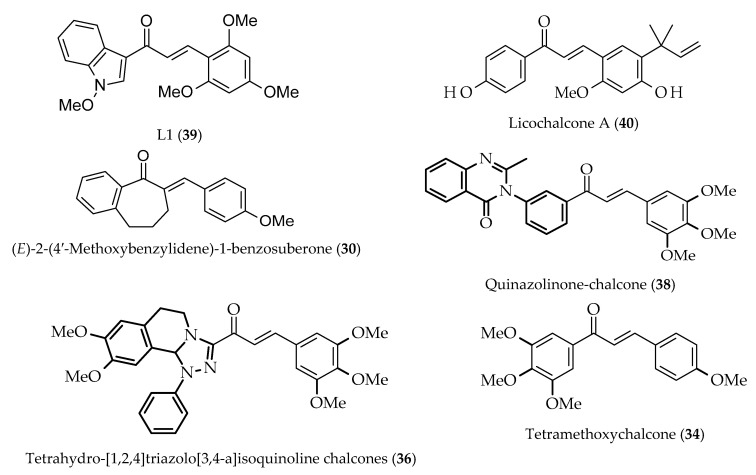
Structures of some chalcones that interfere with the cell cycle.

Studies conducted by Maioral et al. [31] established the importance of chalcone (2*E*)-1-(2,5-dimethoxy-phenyl)-3-(1-naphthyl)-2-propene-1-one (**29**) in blocking the G_2_/M phase in human leukemia cell lines, i.e., NB4, K562, and Kasumi cell lines, and the G_0_/G_1_ phase in the human T-cell leukemia cell line CEM, the human lung cancer cell line U937, and the S phase in Jurkat cell lines. In addition, Kello et al. [32] found that the chalcones (*E*)-2-(4′-methoxybenzylidene)-1-benzosuberone (**30**) and (*E*)-2-(3′,4′-dimethoxy-benzylidene)-1-tetralone (**31**) could be responsible for cell cycle arrest at the G_2_/M phase in Caco-2 cells. Two synthetic chalcones, (*E*)-1-(4-aminophenyl)-3-(2,3-dimethoxyphenyl)-prop-2-en-1-one (**32**) and (*E*)-1-(4-aminophenyl)-3-phenylprop-2-en-1-one) (**33**), were studied for their effects on the cell cycle of the human erythroleukemic cell line K562 and it was observed that both chalcones acted as inhibitors of the cell cycle [33]. The cell cycle distribution of cervical cancer (HeLa) cells treated with the synthetic chalcone L1 (**39**) was analyzed by flow cytometry, revealing its ability to induce cell cycle arrest in the G_2_/M phase after 24 h of treatment [26], while chalcone (**1**) and licochalcone A (**40**) were found to induce the cell cycle arrest in the G_1_ phase in the MCF-7 breast cancer cells [27]. A summary of the effects of different chalcones on the cell cycle is shown in Figure 5, and the structures of chalones that influence the cell cycle are presented in Figure 6. 

### 3.4. Effects on Apoptosis

**Figure 7:**
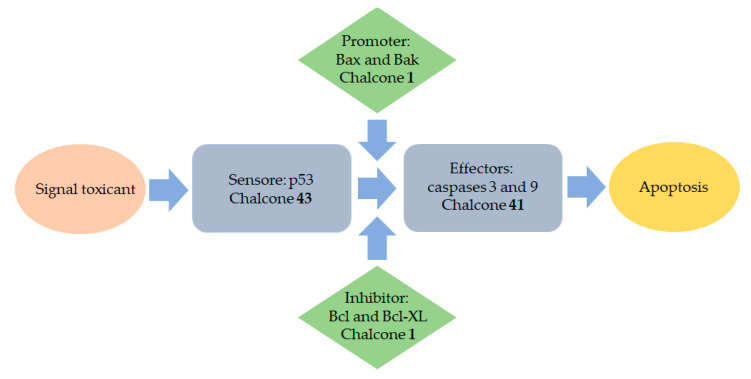
Schematic diagram showing how chalcones induce apoptosis.

Apoptosis is a form of programmed cell death in multicellular organisms, governed by a series of biochemical events [39]. Several studies have examined the effect of chalcones on apoptosis to establish whether chalcones induce cell death through apoptosis or necrosis. One of the early studies reported that chalcones (**1**) could induce apoptosis through enhancing the pro-apoptotic molecules, Bax and Bak, whilst reducing anti-apoptotic Bcl proteins (Figure 7) [40]. Chen et al. [41] demonstrated that the natural chalcone lonchocarpin (**41**) decreased cell proliferation via activation of caspase-3, caspase-9, and Bax (Figure 7). The synthetic chalcone, (2*E*)-3-(2-naphthyl)-1-(3′-methoxy-4′-hydroxy-phenyl)-2-propen-1-one (**42**), could block the G2/M phase, in addition to increasing p53 and Bax expression, thus bringing about caspase-3 activation and cell death [42].

**Figure 8:**
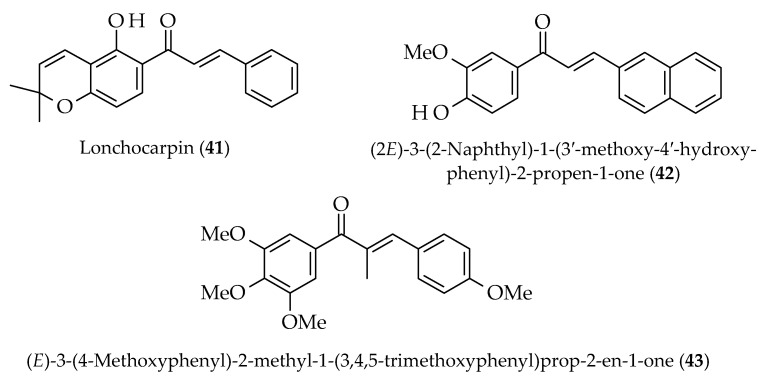
Structures of some chalcones that influence apoptosis.

Furthermore, it was shown that a synthetic chalcone (2*E*)-1-(2,5-dimethoxy-phenyl)-3-(1-naphthyl)-2-propene-1-one (**29**) could induce cell death in human acute leukemia cell lines [31]. A recent study by Novilla et al., (2017) pointed out that synthetic chalcones (*E*)-1-(4-aminophenyl)-3-(2,3-dimethoxy-phenyl)-prop-2-en-1-one (**32**) and (*E*)-1-(4-aminophenyl)-3-phenylprop-2-en-1-one) (**33**) could induce expression of caspase-3 in K562 cells. Additionally, another synthetic chalcone derivative, (*E*)-3-(4-methoxyphenyl)-2-methyl-1-(3,4,5-trimethoxyphenyl)prop-2-en-1-one (**43**), was proposed as useful against prostate cancer as it was noted to cause cell cycle arrest and apoptosis (Figure 7) [43]. The structures of chalones that can influence apoptosis are presented in Figure 8.

### 3.5. Binding with the Estrogenic Receptor (ER)

**Figure 9:**
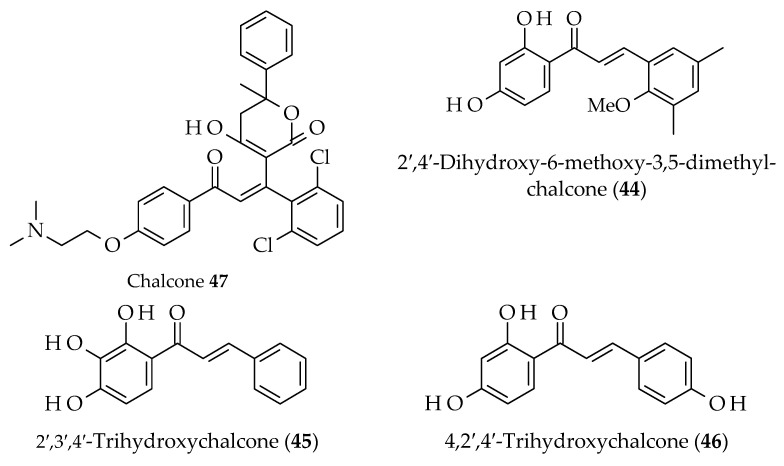
Examples of chalcones that bind to estrogenic receptors.

Chalcones, especially several natural chalcones present in food plants, are known to possess estrogenic properties, which are mediated by their interactions with the estrogenic receptor (ER) [44,45]. Isoliquiritigenin (**22**), a natural chalcone that is usually found in licorice, was shown to have estrogen receptor α-dependent growth-promoting effects on breast cancer cells [44]; its estrogenic effect was established using the hormone-sensitive MCF-7 breast cancer and steroid-independent HeLa cells. This chalcone was found to transactivate the endogenous estrogen receptor α in MCF-7 cells, supported by its ability to induce the down-regulation of estrogen receptor α protein levels and the up-regulation of pS2 mRNA. Furthermore, it was shown that this chalcone could exert agonistic effect for both estrogen receptor isoforms. In silico studies performed with the chalcone 2′,4′-dihydroxy-6-methoxy-3,5-dimethylchalcone (**44**) (Figure 9), a component of the leaves of *Eugenia aquea* established its interaction with estrogen receptor α [45,46]. Another similar virtual screening of 2′,3′,4′-trihydroxychalcone (**45**) showed that this chalcone acted as an estrogen receptor ligand that could modulate the activity of 17β-estradiol [47].

Branham et al. [48] used the estrogen receptor–ligand binding assay to assess the relative binding affinity of five chalcones, i.e., chalcone (**1**), 4-hydroxychalcone (**20**), 4′-hydroxychalcone (**21**), phloretin (**23**), and 4,2′,4′-trihydroxychalcone (**46**), and a significant level of binding with the estrogen receptor α was observed with all these chalcones. In this study, a preliminary attempt was made to describe possible structure–activity relationships of the chalcones used in the study. Chalcone (**1**) was found to bind with the estrogen receptor with a weaker affinity than that of the monohydroxylated chalcones **20** and **21**, whereas multiple hydroxylation, e.g., in chalcone **46**, appeared to have decreased the binding affinity. Several chalcone-phenylpyran-2-one derivatives bearing a *N*,*N*-dimethyl ethylamine side chain, for example, chalcone **47**, were synthesized as estrogen receptor modulators [49]. All synthesized chalcone derivatives showed selectivity toward estrogen receptor α, and among the compounds, the derivative with 2,6-dichloro functionalities (**47**) showed the strongest affinity toward the receptor.

## 4. Bioactivity of Chalcones

**Figure 10:**
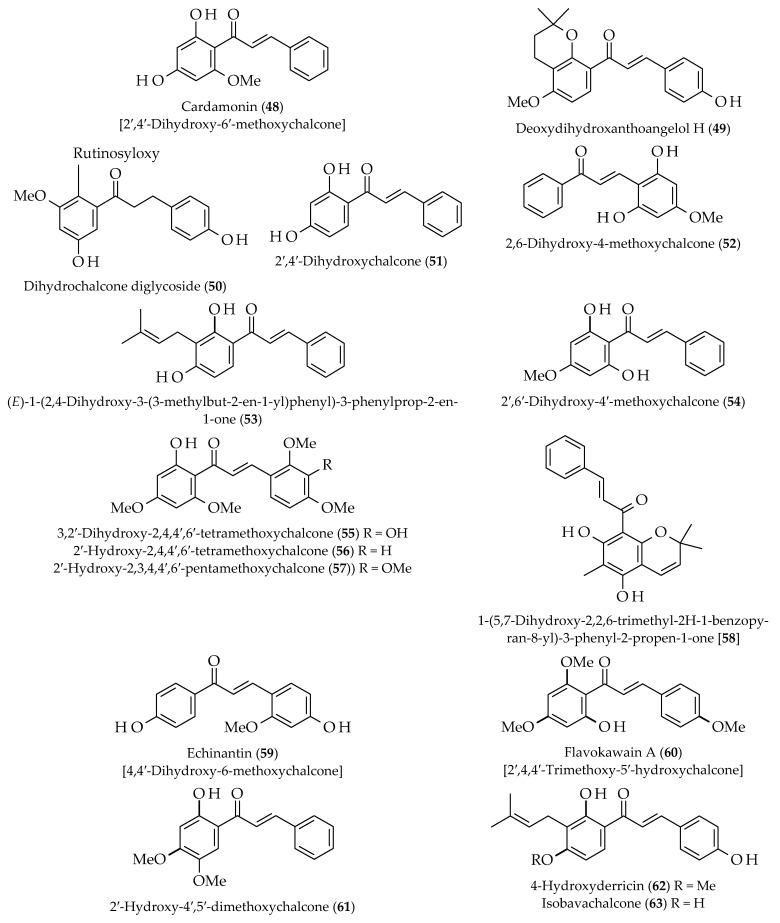

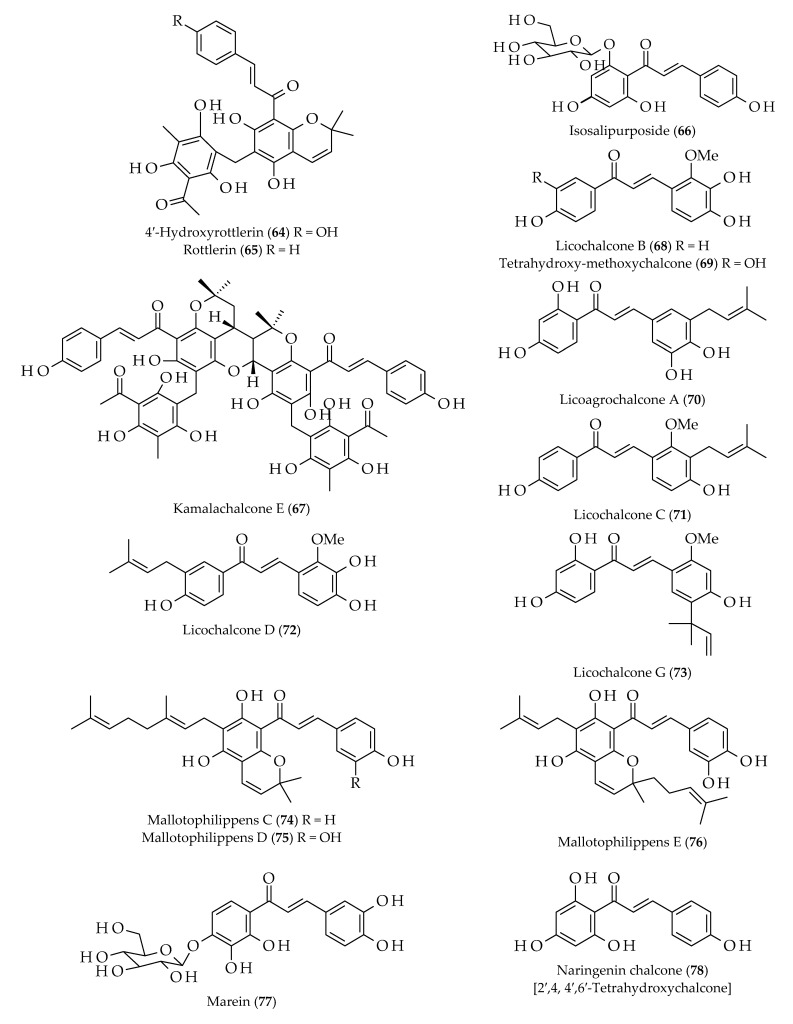

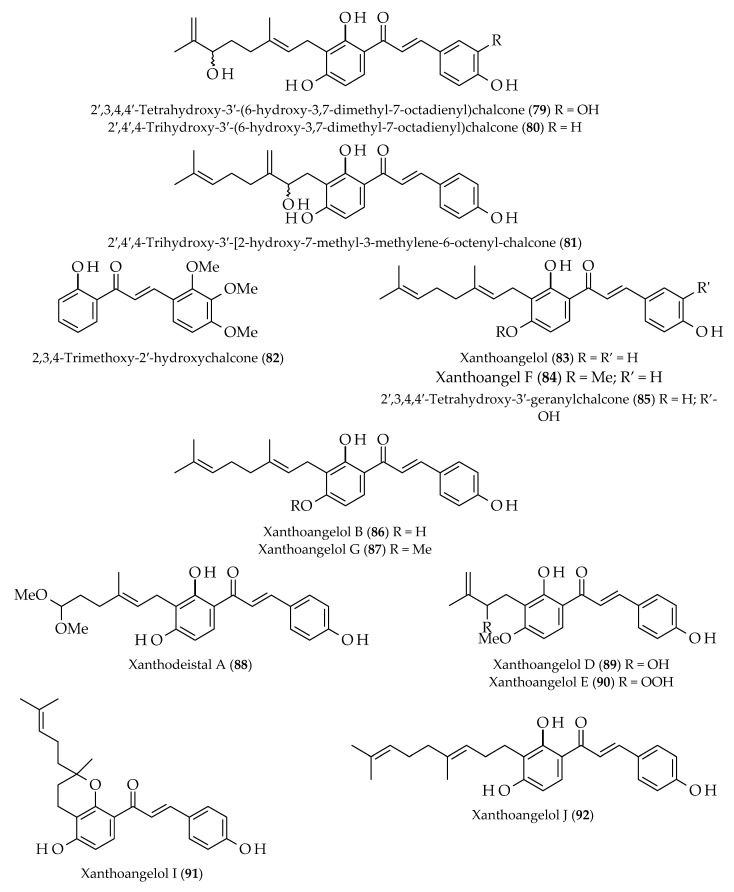
Structures of some naturally occurring bioactive chalcones.

Chalcones are known as biologically active molecules. Different studies with natural and synthetic chalcones have demonstrated their bioactivities in vitro, in vivo, and most recently in silico. Major bioactivities of chalcones include their activities as anticancer, antidiabetic, antimicrobial, and antioxidant agents. Moreover, various chalcones were shown to possess antiparasitic properties. Most of their activities, particularly their anticancer potential, is associated with some of the biomolecular mechanisms outlined in Table 1 in this article. Bioactivities of chalcones are described with specific examples in the following subsections. Figure 10 presents the chemical structures of those bioactive chalcones, unless the structures are shown earlier in this article, whilst Table 2 summarizes various bioactivities of naturally occurring chalcones [50,51,52,53,54,55,56,57,58,59,60,61,62,63,64,65,66,67,68,69,70,71,72,73,74,75,76,77,78,79,80,81].

### 4.1. Anticancer and Cancer Chemopreventive Activity

Several investigations have revealed the bioactivity of chalcones against cancer, either as a cancer chemopreventive agent or as a potential cancer curative agent. However, most of those investigations were *in vitro* or *in silico*. Both natural (Figure 10; Table 2) and synthetic chalcones acted as anticancer agents through the mechanisms sated in Table 1, and mainly the induction of cancer/tumor cell death, predominantly by apoptosis. Research groups who are interested in the cytotoxicity of chalcones, such as Sumiyoshi et al. [59], reported that two chalcone derivatives, xanthoangelol (**83**) and 4-hydroxyderricin (**62**), isolated from *Angelica keiskei* roots could inhibit metastasis and the growth of a tumor. In addition, twenty-one natural chalcones were investigated by Orlikova et al. [22] for their ability to inhibit NF-κB in K562 cells. They found that butein (**16**), calomelanone (**17**), flavokawain C (**18**), homobutein (**19**), 4-hydroxychalcone (**20**), 4′-hydroxychalcone (**21**), isoliquiritigenin (**22**), marein (**77**), 4-methoxychalcone (**2**), and phloretin (**23**) had the ability to inhibit histone deacetylase enzyme (HDAC), and NF-κB.

**Figure 11:**
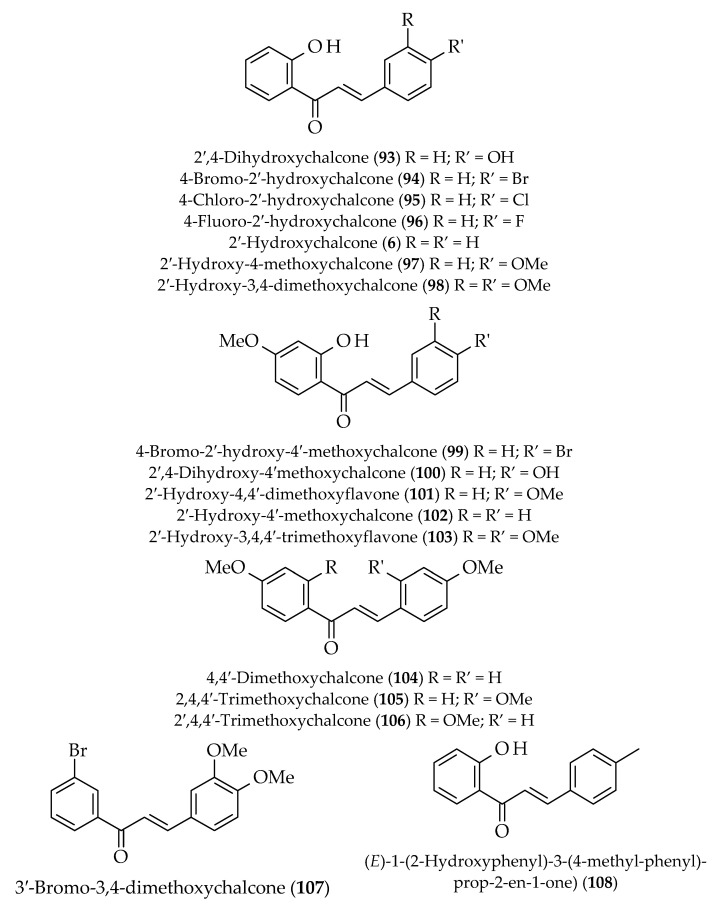
Structures of some synthetic chalcones with anticancer potential.

Inspired by the anticancer potential of naturally occurring chalcones, several synthetic chalcone analogs were produced and assessed for their anticancer properties. For example, Sakagami et al. [82] reported the synthesis of fifteen chalcones (Figure 11), and showed their ability to act as selective anticancer agents in several human oral squamous cancer cell lines, such as HSC-2, HSC-3, HSC-4, and Ca9-22.

Anticancer activity and possible mechanisms of action of 2′-hydroxy-4′,5′-dimethoxychalcone (**61**), isolated from *Sarcandra hainanensis*, were investigated using the lung cancer cell lines, e.g., H322M, H460, H358, H1792 and H157. It was shown that chalcone **61** could increase the level of cellular reactive oxygen species (ROS) and induce cell apoptosis. Ramirez-Tagle et al. [62] used 3′-bromo-3,4-dimethoxychalcone (**107**) and 2,3,4-trimethoxy-2′-hydroxychalcone (**82**) on mouse hepatocytes (HepM) and human hepatoma cells (HepG2 and Huh-7) and found that both chalcones could lead to cellular apoptosis and to an increase ROS levels. Synthetic chalcone (*E*)-1-(2-hydroxyphenyl)-3-(4-methyl-phenyl)-prop-2-en-1-one (**108**) was identified as a chemopreventive agent with the results indicating that it could decrease the extent of DNA damage [83].

### 4.2. Antidiabetic Activity

Some naturally occurring chalcones were shown to possess antidiabetic properties. For example, 2′,6′-dihydroxy-4′-methoxychalcone (**52**), isolated from the flowers of *Piper claussenianum* [63], and 4-hydroxyderricin (**62**), and xanthoangelol (**83**) from *Angelica keiskei*, were found to have antidiabetic properties [64]. Chalcone **52** exhibited its antidiabetic properties through producing a hypoglycemic effect in streptozotocin-induced diabetes in rats. It was observed that this compound could reduce blood glucose levels from 277.4 mg/dL before treatment to 158.8 mg/dL after 12 days of treatment. Both 4-hydroxyderricin (**62**) and xanthoangelol (**83**) displayed insulin-like behavior via a pathway that is not dependent on the activation of peroxisome proliferator-activated receptor-γ [64], whereas chalcone **62** could prevent the progression of diabetes in genetically diabetic KK-A^y^ mice.

**Figure 12:**
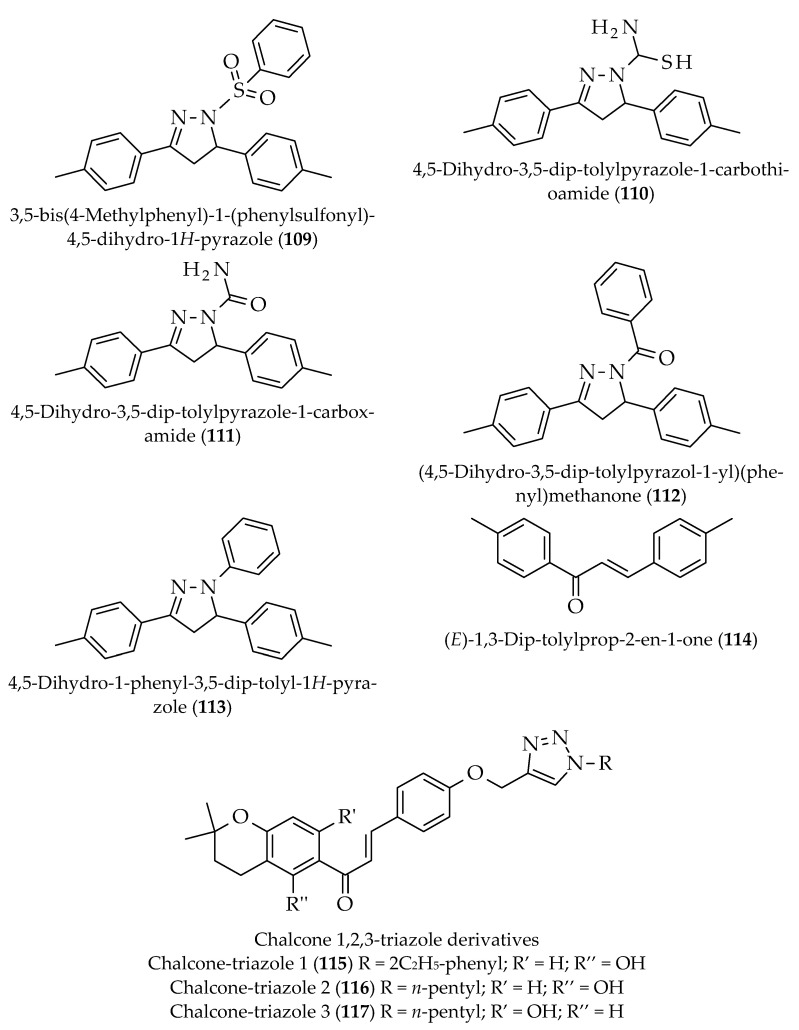
Structures of some synthetic antidiabetic chalcones.

The antidiabetic effect of naturally occurring chalcones prompted several bioinspired syntheses of chalcone analogs, which were subsequently tested for their antidiabetic potential. For instance, synthetic chalcones, (*E*)-1,3-dip-tolylprop-2-en-1-one (**114**), 4,5-dihydro-3,5-dip-tolylpyrazole-1-carbothioamide (**110**), 4,5-dihydro-3,5-dip-tolylpyrazole-1-carboxamide (**111**), (4,5-dihydro-3,5-dip-tolylpyrazol-1-yl)(phenyl)methanone (**112**), 4,5-dihydro-1-phenyl-3,5-dip-tolyl-1*H*-pyrazole (**113**), and 3,5-bis(4-methylphenyl)-1-(phenylsulfonyl)-4,5-dihydro-1*H*-pyrazole (**109**), were shown to act as hypoglycemic agents by decreasing the level of blood glucose in alloxan-induced diabetic rats [84]. Among these chalcone derivatives, 4,5-dihydro-1-phenyl-3,5-dip-tolyl-1*H*-pyrazole (**113**) produced the most prominent antidiabetic effect. In addition, Chinthala et al. [85] synthesized twenty chalcone derivatives, three of which (**115**–**117**) (Figure 12) were found to exert α-glucosidase-inhibitory activity (IC_50_ = 67.7–102.1 μM). Based on the obtained results with these chalcone derivatives, it was assumed that the presence of aliphatic chain length up to five carbons (*n*-pentyl) attached to the triazole ring might be optimum for generating α-glucosidase activity.

### 4.3. Anti-Inflammatory Activity

Naturally occurring chalcones are phenolic compounds, and often possess one or more phenolic hydroxyl functionality in their structures, which generally offer them with the inherent free-radical-scavenging properties that can be useful against oxidative stress. It is known that oxidative stress is associated with inflammatory responses. Thus, any reduction in oxidative stress is expected to inhibit inflammatory responses. Chalcones with free-radical-scavenging properties were shown to also possess anti-inflammatory properties. The examples of naturally occurring anti-inflammatory chalcones could include flavokawain B (**28**) and isoliquirigenin (**22**) from *Alpinia pricei* [65], licoagrochalcone A (**70**) and licochalcone B (**68**) from *Glycyrrhiza inflata* [66], licochalcone C (**71**) from *Glycyrrhiza glabra* [67], and mallotophilippens C-E (**74**–**76**) from *Mallotus philippinensis* [68]. It was shown that flavokawain B (**28**) could inhibit the production of nitric oxide (NO) and prostaglandin E_2_ (PGE2) in lipopolysaccharide (LPS)-induced murine leukemia macrophage RAW 264.7 cells [65]. Furthermore, this chalone dose-dependently decreased the secretion of TNF-R and inhibited the expression of iNOS (inducible nitric oxide synthase) and COX2 (cyclooxygenase 2) proteins. Flavokawain B (**28**) was also found to block the nuclear translocation NF-κB and thus decrease NF-κB protein levels in the nucleus. Similar anti-inflammatory activity was observed with the chalcone **28** in vivo using a mouse model, where the pre-administration of a 200 mg/kg compound could reduce the NO concentration and significantly suppress LPS-induced iNOS, COX-2, and NF-κB proteins expression in mouse liver. Licoagrochalcone A (**70**) and licochalcone B (**68**) showed prominent anti-inflammatory activity with IC_50_ values of 9.35 and 8.78 μM, respectively, on LPS-induced NO production. Additionally, chalcone **70** showed potent inhibitory effect on NF-κB transcription [66]. Mallotophilippens C-E (**74**–**76**) were found to inhibit NO production in activated RAW 264.7 cells and the expression of iNOS, COX2, IL-6 (interleukin 6), and IL-1β mRNA [68], suggesting that these compounds might exert their anti-inflammatory properties through inactivation of NF-κB.

**Figure 13:**
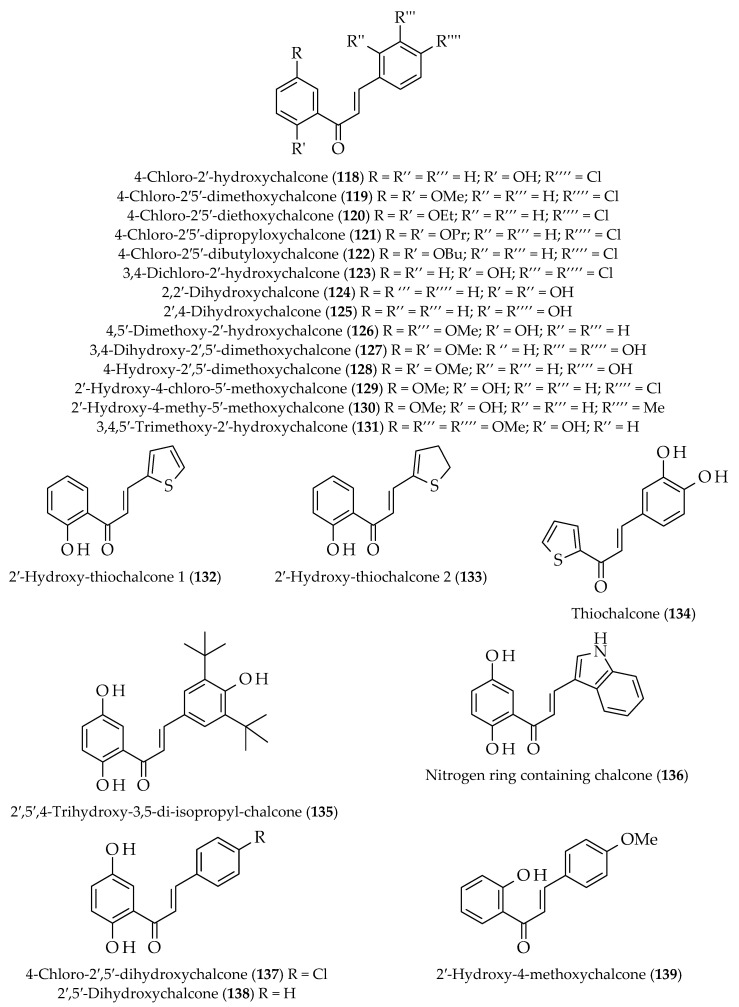
Structures of some synthetic anti-inflammatory chalcones.

Anti-inflammatory properties of naturally occurring chalcones, as exemplified above, prompted bioinspired synthesis of several chalcone analogs as anti-inflammatory agents [86,87,88,89]. Over two decades ago, several 2′-hydroxychalcones and 2′,5′-dihydroxychalcones (Figure 13) were synthesized and their anti-inflammatory properties were ascertained by observation of their in vitro inhibitory effects on the activation of mast cells, neutrophils, microglial cells, and macrophages [86]. Among these synthetic chalcones, 2,2′-hydroxychalcone (**124**) emerged as the most potent anti-inflammatory agent that could inhibit the release of β-glucuronidase (IC_50_ = 1.6 μM) and lysozyme (IC_50_ = 1.4 μM) from rat neutrophils. Later, eleven chalcone derivatives (Figure 13) were synthesized by the Claisen–Schmidt condensation of acetophenones and aromatic aldehydes, or produced with suitable dihydrochalcone and alkyl bromide, or prepared in one-pot synthesis involving acetophenone and aromatic aldehyde under ultrasonication [87], and their anti-inflammatory potential was assessed. Chalcone derivatives **125**, **132**, **133**, and **136** showed a considerable level of inhibition on the release of β-glucuronidase or lysozyme from rat neutrophils stimulated with appropriate stimuli; chalcones **125** and **133** inhibited superoxide anion production in rat neutrophils; and chalcones **123**, **128**, and **135** could inhibit NO production. Jantan et al. [88] reported the synthesis of chalcone derivatives and showed their potential as inhibitors of secretory phospholipase A2, cyclooxygenases, lipoxygenase, and pro-inflammatory cytokines by a series of in vitro enzyme inhibition and in silico assays. Chalcone derivatives with 4-methylamino-ethanol functionality appeared to be the most potent ones. Most recently, a synthetic chalcone (**139**) (Figure 13) was demonstrated to possess antioxidant, anti-inflammatory, and neuroprotective properties [89]. This chalcone could attenuate LPS-induced inflammation in RAW 264.7 macrophages.

### 4.4. Antimicrobial Activity

There are several reports available in the literature describing the antimicrobial activity of natural and synthetic chalcones; these activities include their activity against several pathogenic bacterial, fungal, and viral species. Figure 10 and Table 2 present some examples of naturally occurring antimicrobial chalcones. Here, the antimicrobial activity of chalcones is discussed under three subsections: antibacterial, antifungal, and antiviral chalcones.

#### 4.4.1. Antibacterial Activity

Antibacterial activity of naturally occurring chalcones is well-documented in the literature [69,70] (Table 2). For example, 2′,4′-dihydroxychalcone (**51**) isolated from *Zuccagnia punctata*; 3,2′-dihydroxy-2,4,4′,6′-tetramethoxychalcone (**55**), 2′-hydroxy-2,4,4′,6′-tetramethoxychalcone (**56**), and 2′-hydroxy-2,3,4,4′,6′-pentamethoxychalcone (**57**) from *Piper hispidum*; and isoliquiritigenin (**22**) from the Brazilian propolis (*Apis melifera*) were shown to possess antibacterial properties [69,70,71]. 2′,4′-Dihydroxychalcone (**51**) was found active against the Gram-positive and Gram-negative bacterial species, including *Acinetobacter baumannii*, *Enterobacter cloacae*, *Morganella morganii*, *Proteus mirabilis*, *Pseudomonas aeruginosa*, *Serratia marcescens,* and *Stenotrophomonas maltophilia*, which have MIC values ranging between 0.10 μg/mL and 100 μg/mL [69]. 3,2′-Dihydroxy-2,4,4′,6′-tetramethoxychalcone (**55**), 2′-hydroxy-2,4,4′,6′-tetramethoxychalcone (**56**) and 2′-hydroxy-2,3,4,4′,6′-pentamethoxychalcone (**57**) were shown active against *Staphylococcus aureus* with MIC values in the range of 125–250 μg/mL [70], whilst the antibacterial activity isoliquiritigenin (**22**) was observed against *Actinomyces naeslundii*, *Staphylococcus aureus*, and *Streptococcus mutans* (MIC = 15.6–62.5 μg/mL) [71].

**Figure 14:**
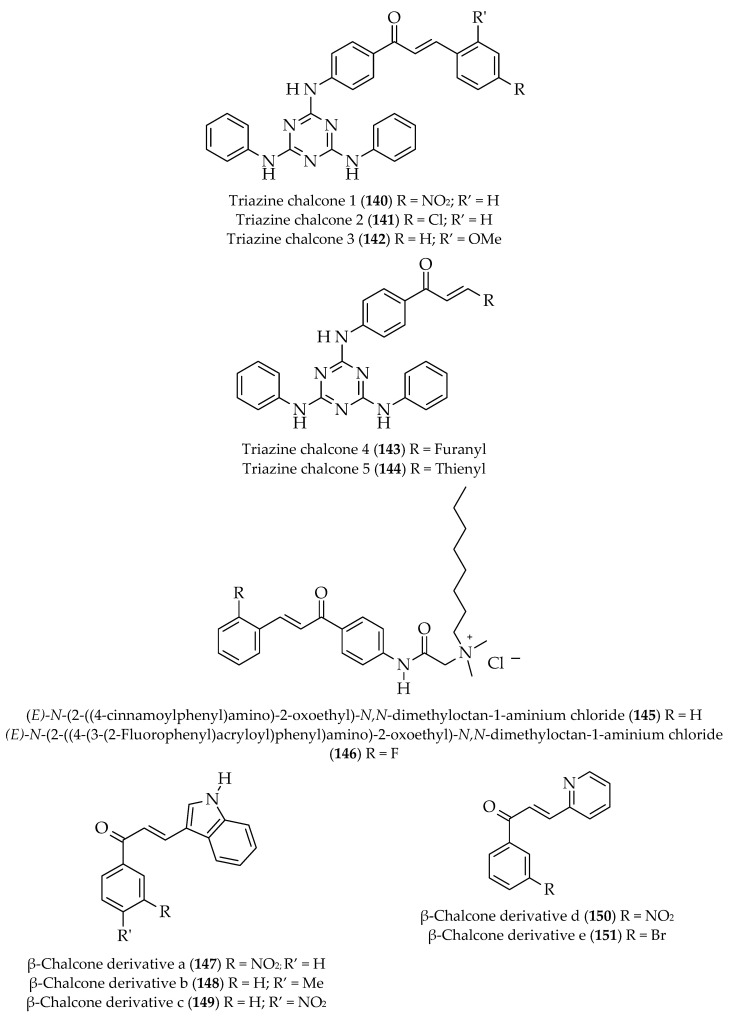

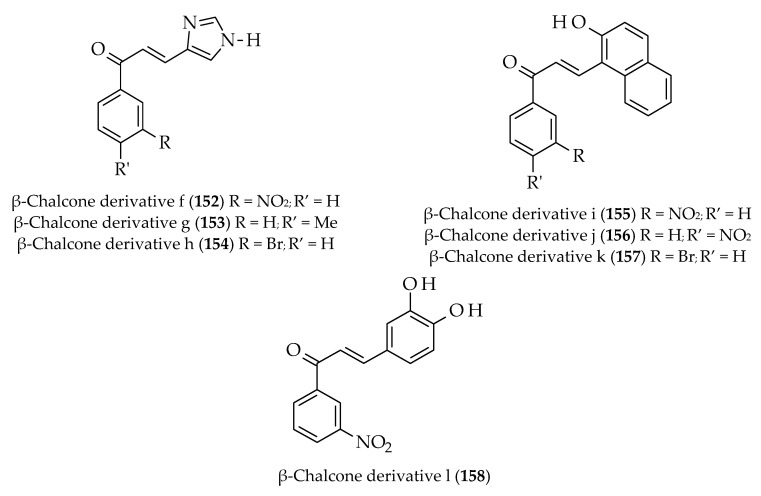
Structures of some synthetic antibacterial chalcones.

The antibacterial activity of natural chalcones inspired synthetic chemists to attempt bioinspired synthesis of antibacterial chalcone derivatives. For example, a decade ago, Solankee et al. [90] investigated synthetic triazine-based chalcones (Figure 14) as antibacterial agents, and reported inhibition of the growth of *Bacillus cereus*, *Enterobacter cloacae*, *Escherichia coli*, *Listeria monocytogenes*, *Micrococcus flavus*, *Pseudomonas aeruginosa*, *Salmonella typhimurium*, and *Staphylococcus aureus*. However, except for the furanyl derivative (**143**), the activity of other compounds was rather weak. Chu et al. [91] resynthesized 29 cationic chalcones with varying aryl substitutions, and diversity in the *N*,*N*-dimethyl alkylamine length, and tested all compounds against *Enterococcus faecalis, Escherichia coli, Salmonella enterica* and *Staphylococcus aureus*. Chalcone derivatives, (*E*)-*N*-(2-((4-cinnamoylphenyl)amino)-2-oxoethyl)-*N*,*N*-dimethyloctan-1-aminium chloride (**145**) and (*E*)-*N*-(2-((4-(3-(2-fluorophenyl)acryloyl)phenyl)amino)-2-oxoethyl)-*N*,*N*-dimethyloctan-1-aminium chloride (**146**) (Figure 14), demonstrated a considerable level of growth inhibitory activity against both Gram-negative and Gram-positive bacteria, including the drug-resistant New Delhi metallo-β-lactamase-1 (NDM), *Klebsiella pneumoniae* carbapenemase (KPC), and methicillin-resistant *Staphylococcus aureus* (MRSA) strains. While (*E)*-*N*-(2-((4-cinnamoylphenyl)amino)-2-oxoethyl)-*N*,*N*-dimethyloctan-1-aminium chloride (**145**) was most active against *E. coli* (MIC = 2 μg/mL), derivative **146** showed the most prominent activity against *S. aureus* (MIC = 0.5 μg/mL). This finding demonstrated the potential applications of chalcone peptidomimetics as a new class of antibacterial agents.

Most recently, a series of β-chalcone derivatives (**147**–**158**) (Figure 14) was synthesized using various substituted amines by the Claisen-Schmidt condensation reaction in basic condition, and evaluated for antibacterial activity against different species of bacteria, including *Enterococcus faecalis*, *Escherichia coli*, *Klebsiella pneumoniae*, *Pseudomonas aeruginosa*, *Salmonella enterica* and *Staphylococcus pneumoniae* [92]. Among these synthetic chalcone derivatives, compound **148** emerged as the most potent antibacterial agent which was particularly effective against *E. coli* (MIC = 125 μg/mL). *In silico* studies established DNA binding and molecular docking of these compounds. There are several other examples of synthesis antibacterial chalcones and assessment of their modes of action available in the literature [93,94,95,96,97].

#### 4.4.2. Antifungal Activity

Chalcones are known to possess antifungal properties. One of the early studies on antifungal activities of naturally occurring chalcones was conducted by Jayasinghe et al. [73] (Table 2). In that work, several chalcones (**79**–**81**, **83** and **85**) were isolated from the leaves of *Artocarpus nobilis*, and assessed for fungicidal activities against *Cladosporium cladosporioides*. All those chalcones showed fungicidal activity (2–15 μg/spot) in the thin layer chromatography (TLC) bio-authography method. 1-(5,7-Dihydroxy-2,2,6-trimethyl-2H-1-benzopyran-8-yl)-3-phenyl-2-propen-1-one [58], 4′-hydroxyrottlerin (**64**), kamalachalcone E (**67**) and rottlerin (**65**), isolated from the fruits of *Mallotus philippinensis*, were tested for antifungal activity against several human pathogenic fungus [72]. Chalcone **58** and the chalcone dimer **67** showed significant antifungal properties (IC_50_ = 4–16 μg/mL) against *Aspergillus fumigates* and *Cryptococcus neoformans*. Both compounds were particularly effective against *Cryptococcus neoformans* (IC_50_ = 4 μg/mL). However, none of those chalcones displayed any noticeable antifungal activity against *Aspergillus flavus*, *A. niger,* *Candida albicans, C. glabrata*, and *C. tropicalis* [72]. 3,2′-Dihydroxy-2,4,4′,6′-tetramethoxychalcone (**55**), 2′-hydroxy-2,4,4′,6′-tetramethoxychalcone (**56**), and 2′-hydroxy-2,3,4,4′,6′-pentamethoxychalcone (**57**) were shown active against *C. albicans* with MIC values in the range of 250-500 μg/mL [70].

**Figure 15:**
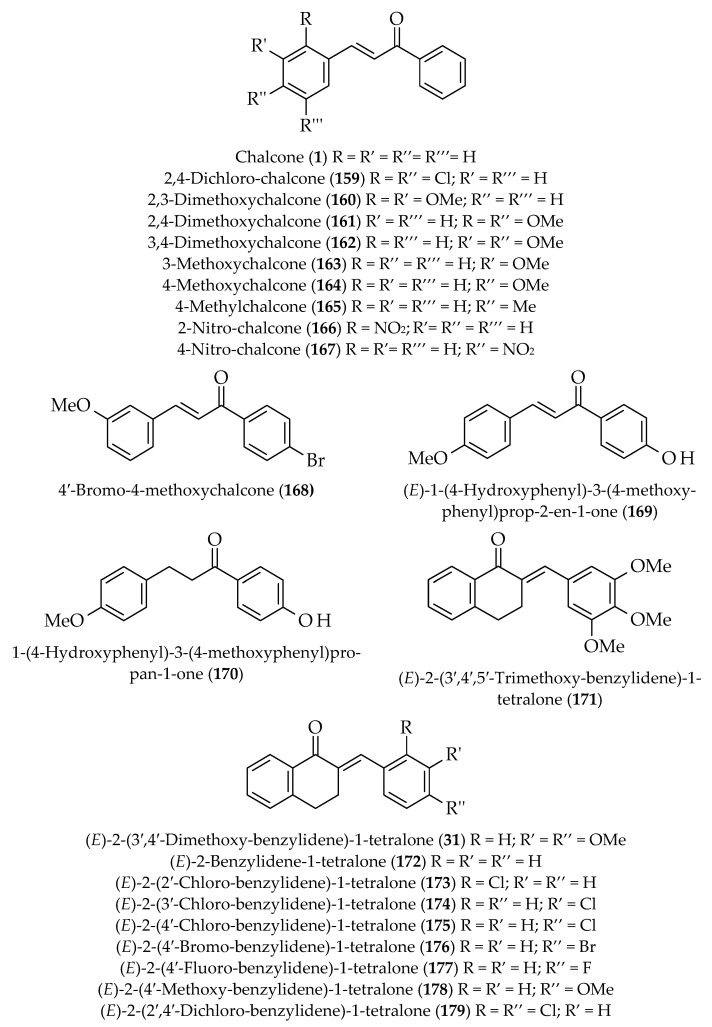
Structures of some synthetic antifungal chalcones.

These initial findings on antifungal properties of natural chalcones led the way to bioinspired synthesis of several other chalcone analogs with antifungal activities. Just over two decades ago, Lopez et al. [98] synthesized 41 chalcone derivatives, 11 of which (**1**, **159**–**168**) (Figure 15) were found active against dermatophytes, e.g., *Epidermophyton* *floccosum*, *Microsporum canis, M. gypseum, Trichophyton mentagrophytes* and *T. rubrum* (MIC = 1.5–12.5 μg/mL) These chalcones (**1**, **159**–**168**) showed inhibitory properties against polymers of the fungal cell wall. However, none of these chalcones was active against *Aspergillus niger, A. fumigatyus, A. flavus, Candida albicans, Cryptococcus neoformans* and *Saccharomyces cerevisiae*. Antifungal activity of 12 further synthetic chalcones, albeit some of them are actually natural chalcones, were tested against *Penicillium chrsogenum*, *A. niger, A. fluvus* and *Anthrobortys oligospora*, and (*E*)-1-(4-hydroxyphenyl)-3-phenylprop-2-en-1-one (**21**), were found active against *P. chrsogenum* and *A. nigar*, (*E*)-1-(4-hydroxyphenyl)-3-(4-methoxyphenyl)prop-2-en-1-one (**169**) displayed fungicidal effect against *P. chrsogenum* and 1-(4-hydroxyphenyl)-3-(4-methoxyphenyl)propan-1-one (**170**) was active against *A. niger* [99] (Figure 15).

Gupta et al. [100] synthesized 10 chalcone derivatives (**31**, **171**–**177**) (Figure 15) and tested for their antifungal properties. None of these chalcones were active against *A. niger* and *C. albicans*, but showed strong fungicidal activity (MIC = 1.5–50.0 μg/mL) against the dermatophyte *Microsporum gypseum*. It is noteworthy that (*E*)-2-benzylidene-1-tetralone (**172**) and (*E*)-2-(4′-chloro-benzylidene)-1-tetralone (**175**) exhibited better antifungal activity than the positive control ketoconazole, a well-known antifungal drug. It appeared that the addition of a Cl functionality at C-4′ of these compounds could enhance the antifungal potency. There are several other publications available to date that describe the synthesis and antifungal activity of chalcone derivatives having versatile chemical features [101,102,103,104].

#### 4.4.3. Antiviral Activity

Chalcones were demonstrated to possess antiviral properties, which is thought to be exerted mainly by the disruption of the different stage of viral replication cycle, the inhibition of viral or cell enzymes, and/or the induction of apoptosis. One of the earliest studies on the antiviral activities of naturally occurring chalcones was conducted about two decades ago by Ochiumi et al. [76] (Table 2). In that study, naturally occurring chalcones, including licochalcone A (**40**), licochalcone B (**68**), and tetrahydroxy-methoxychalcone (**69**), were found to suppress TPA (12-*O*-tetradecanoylphorbol-13-acetate)-induced HIV promoter activity, and it was suggested that these chalcones could be used as templates for the development of novel anti-HIV drugs. Later, licochalcone A (**40**), licochalcone D (**72**), and licochalcone G (**73**), isolated from *G. inflata*, were reported to possess antiviral properties against influenza virus [75]. Similarly, echinantin (**59**) and isoliquiritigenin (**22**) from the same plant were also active against this virus. The antiviral activity was measured by their noncompetitive neuraminidase inhibitory activity. Among these chalcones, echinantin (**59**) was the most potent one with an IC_50_ value in the range of 2.19–5.80 μg/mL against various strains of the influenza virus, e.g., H1N1, H9N2, novel H1N1 (WT), and oseltamivir-resistant novel H1N1 (H274Y), expressed in 293T cells. In another study, dihydrochalcone diglycoside (**50**), isolated from the seagrass *Thalassodendrin ciliatum*, showed antiviral activity against the influenza A virus [74], and is assumed to have resulted from the electronic interactions between atomic charges within this compound in both aromatic rings and *pseudo*-receptor structures in the cells which prevent the attachment and penetration of the virus to the cell. Several alkylated chalcones, including xanthoangelol (**83**), xanthoangelols B, D-G (**84**–**87**), and xanthodeistal (**88**), isolated from *Angelica keiskei* (Table 2), were shown to possess significant antiviral properties [77], as evident from their inhibitory activity against cysteine proteases of SARS-CoV viruses. Among these compounds, xanthoangelol E (**90**), containing the perhydroxyl group, exhibited the most potent inhibitory activity against SARS-CoV 3CL^PRO^ and PL^PRO^ with IC_50_ values of 11.4 and 1.2 μM.

**Figure 16:**
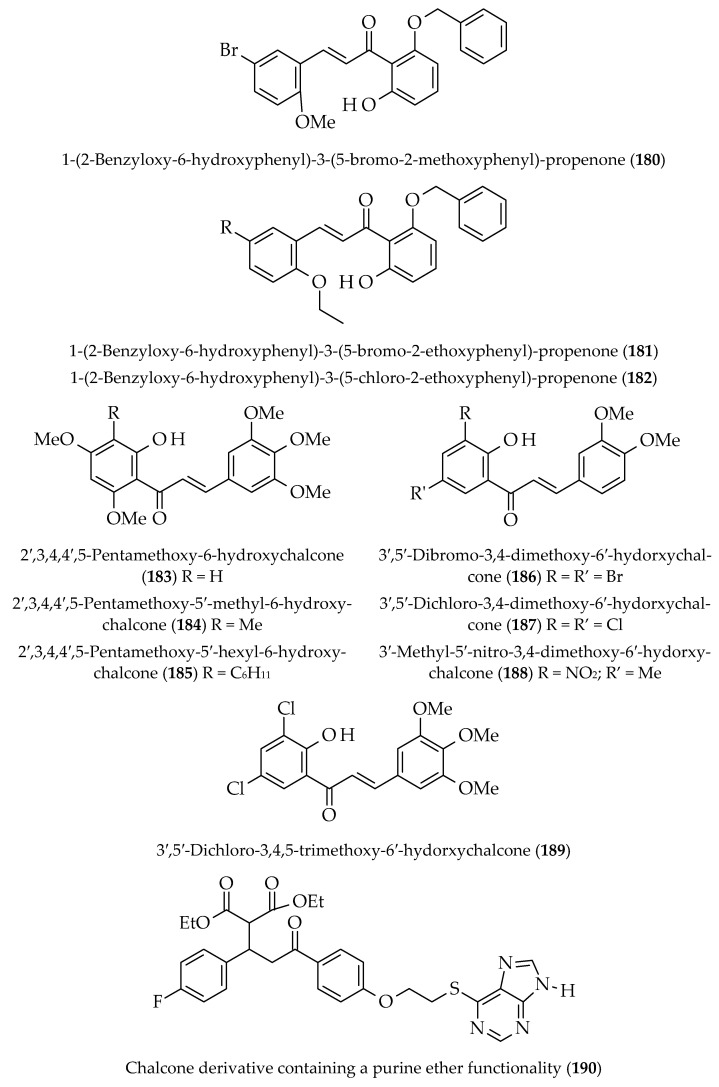
Structures of some synthetic antiviral chalcones.

Promising antiviral properties of various naturally occurring chalcones tempted synthetic chemists to indulge in bioinspired synthesis of chalcone derivatives with potential antiviral properties. Such efforts have recently been further intensified with the emergence of various deadly respiratory viruses, and viral diseases including the current COVID-19 pandemic. Several studies have identified the activity of synthetic chalcones against different viruses. For example, Cole et al. [105] synthesized several *O*-benzyl-substituted chalcones, e.g., 1-(2-benzyloxy-6-hydroxyphenyl)-3-(5-bromo-2-methoxyphenyl)-propenone) (**180**), and determined their antiviral properties, suggesting that these compounds could be used for the prevention or treatment of human immunodeficiency virus (HIV) infections, whilst Mateeva et al. [106] investigated several synthetic chalcones (**183**–**189**) (Figure 16) as antiviral agents, and showed that all chalcones at a concentration of 5 μM could inhibit, albeit at variable potencies, the translation of hepatitis C virus. The inhibition of phosphorylated rsp6 (ribosomal protein S6) and S6K1 (ribosomal protein S6 kinase beta-1) by these chalcones could dampen hepatitis C virus translation. This inhibition potentially was mediated through inhibition of mTOR (mechanistic target of rapamycin), which is a protein kinase that regulates cell growth, survival, and metabolism.

1-(2-Benzyloxy-6-hydroxyphenyl)-3-(5-bromo-2-methoxyphenyl)-propenone (**180**) was found to be the most potent as an antiviral agent, and could inhibit several clinical isolates of HIV in a dose-dependent fashion with IC_50_ values of around 5 μM [105]. This finding led to further modification of this structure (**180**) to enhance antiviral potency and reduce toxicity towards normal human cells, which resulted in the synthesis of two more antiviral chalcones (**181**) and (**182**). Both chalcones (**181** and **182**) at a concentration of 10 μM offered >92% inhibition of viral propagation without impacting host cell viability. It appeared that halogenation at C-5 is an essential requirement for the activity of these benzoyloxychalcones. An ethoxy substituent in (**181**) and (**182**), instead of a methoxy in **180**, offered increased efficacy, and a balance between high antiviral potency and low toxicity to host cells.

Most recently, Fu et al. [107] synthesized 28 chalcone derivatives containing a purine ether functionality, and derivative (**190**) (Figure 16) emerged as the best candidate to effectively inhibit the infectivity of tobacco mosaic virus in vivo with an EC_50_ value of 65.8 μg/mL. It was demonstrated that this chalcone derivative could destroy the integrity of tobacco mosaic virus. A docking study suggested that the antiviral activity of (**190**) might depend on its strong binding affinity to tobacco mosaic virus coat protein (TMV-CP). There are several other reports on antiviral properties of synthetic chalcone derivatives available in the literature to date, including a few excellent review articles on this topic [108,109,110].

### 4.5. Antioxidant Activity

Antioxidants are compounds that can stop or prevent oxidative processes. They can be natural compounds as well as synthetic products. Antioxidants are effective in the management of oxidative stress, which is considered as one of the major causes of several chronic and life-threatening diseases. External supply of antioxidants is important for human health, and often achieved through regular diets containing plenty of fruits and vegetables. However, there are times when the additional supply of antioxidants in formulated forms may become essential. Plants have long been known as a source of antioxidants, and the phenolic and polyphenolic compounds are a major class of natural antioxidants. Most of the naturally occurring chalcones are phenolic compounds, which possess significant free-radical-scavenging and antioxidant properties. For example, lichochalcone C (**71**) [67] from *Glycyrrhiza glabra* and naringenin chalcone (**78**) and its glucoside, isosalipurposide (**66**), from *Acacia* *cyanophylla* [50] were shown to possess antioxidant properties (Table 2). Similarly, several chalcones (**79**–**81**, **83**, and **85**) were isolated from the leaves of *Artocarpus nobilis*, and were demonstrated to have antioxidant activity [73]. The antioxidant activity of isosalipurposide (**66**) was established by the 1,1-diphenyl-2-hydrazyl (DPPH) assay, the 2,2′-azino-bis(3-ethylbenzothiazoline-6-sulfonate) (ABTS) cation radical-scavenging assay, and the reducing power assay. The aglycone of glycoside **66**, commonly known as naringenin chalcone (**78**), could have enhanced antioxidant potency because of the presence of an additional phenolic hydroxyl group. Additionally, this chalcone (**66**) was shown active against acetylcholinesterase with an IC_50_ value of 52.04 μg/mL [50]. Lichochalcone C (**71**) at a concentration of 50 μM was found to significantly influence the antioxidant network activity of superoxide dismutase (SOD), catalase (CAT) and glutathione peroxidase (GPX) activity [67]. The geranylated chalcones (**79**–**81**, **83**, and **85**) showed strong qualitative radical-scavenging activity in the TLC-based DPPH assay [73].

**Figure 17:**
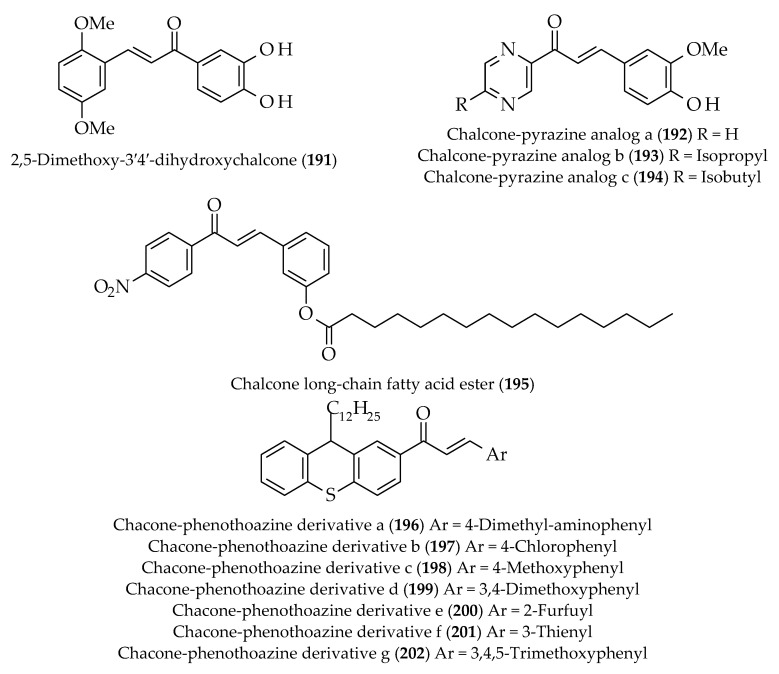
Structures of some synthetic antioxidant chalcones.

Both the naturally occurring chalcones, and several synthetic chalcones, and their derivatives, were shown to possess antioxidant properties of varied potencies based on structural variations and diversity. Most recently, from a series of synthetic β-chalcone derivatives, chalcone (**148**) (Figure 14) was studied for its antioxidant activity using the DPPH and hydrogen-peroxide (H_2_O_2_) assays [92]; this chalcone showed significant antioxidant (radical-scavenging) activity with the IC_50_ values of 298 and 530 μg/mL, respectively, in the DPPH and H_2_O_2_ assays. Wang et al. [111] synthesized 41 chalcone derivatives and assessed their dual antioxidant mechanisms. Among the compounds, chalcone **191** (Figure 17) was found to be the most potent one, and showed cytoprotection of H_2_O_2_-induced oxidative damage in phaeochromocytoma (PC12) cells through free-radical-scavenging (as confirmed from the DPPH assay) and activation of the Nrf2/ARE antioxidant pathway at the same time. This chalcone (**191**) also displayed a noticeable effect against ischemia/reperfusion-related brain injury in animals. It was suggested that 2,5-dimethoxy-3′4′-dihydroxychalcone (**191**) could act through dual antioxidant mechanisms, and could be considered as a direct and indirect antioxidant. A total of 26 chalcone derivatives with alkyl-substituted pyrazine heterocycle were synthesized and assessed for their radical-scavenging as well as cellular antioxidant capacity, impacting the growth of cells exposed to H_2_O_2_ [112]. Three of these synthetic chalcone analogs (**192**–**194**) (Figure 17) exhibited DPPH-radical-scavenging activity (IC_50_ = 39–186 μM) through a single electron transfer followed by a proton transfer mechanism, as revealed in the density functional theory (DFT) modeling. Clearly, chalcone **193** was the most active free-radical-scavenger with an IC_50_ value of 39 μM in the DPPH assay. Earlier, Lahsasni et al. [113] synthesized a library of chalcone derivatives and chalcone **195** emerged as the most potent antioxidant among the analogs, and the activity was better than that of the well-known antioxidant ascorbic acid in the DPPH assay.

2′-Hydroxy-4-methoxychalcone (**139**), a recently synthesized chalcone, showed a considerable antioxidant activity [89]. It was found to be able to attenuate LPS-induced oxidative stress and promote antioxidant defense in RAW264.7 macrophages. 2′-Hydroxy-4-methoxychalcone pretreatment at a concentration range of 0.01-–1.0 μM could augment the nuclear expression of Nrf2 in a concentration-dependent manner. An increased level of the downstream antioxidant protein heme oxygen SE-1 was also observed. Additionally, this pretreatment was found to significantly increase the levels of non-enzymatic antioxidant GSH (glutathione) in LPS-stimulated RAW 264.7 cells. Zahrani et al. [114] reported the synthesis of several chalcone-based phenothiazine derivatives and their antioxidant potentials based on the DPPH assay; seven of these compounds (**196**–**202**) showed promising DPPH-radical-scavenging activity. There are several other reports depicting the synthesis and antioxidant properties of chalcone derivatives available in the literature, most of which possess DPPH-radical-scavenging properties [115,116,117,118].

### 4.6. Antiparasitic Activity

Parasites are organisms that live and feed on another living being, e.g., animals, humans, insects, or plants, and most often cause harm to the host organism. Parasitic diseases, such as like leishmaniasis and malaria, remain a major global health concern, and research consequently continues in the search for effective and affordable drugs to combat various parasitic diseases. Some naturally occurring chalcones were shown to possess antiparasitic activities (Table 2). For example, flavokawain B (**28**), isolated from Polygonum ferrugineum [79]; (*E*)-1-(2,4-dihydroxy-3-(3-methylbut-2-en-1-yl)phenyl)-3-phenylprop-2-en-1-one (**53**) from *Lonchocarpus* sp. [78]; licoagrochalcone A (**70**) from *Erythrina abyssinica* [81]; and licochalcone C (**71**) isolated from *Glycyrrhiza glabra* [80] were found to possess significant antiparasitic/antiprotozoal properties. A protozoa can be free-living or parasitic. Most of the disease-causing protozoa are parasitic. Even free-living protozoa when entering other cells and tissues of living being become parasitic. While licochalcone C (**71**) was found active against malarial parasite *Plasmodium falciparum*, chalcone **53** showed activity against *Leishmania* and *Trypanosoma* species. Flavokawain B (**28**) demonstrated trypanocidal activity against *Trypanosoma cruzi* and *T. bruceii*, with IC_50_ values of 9.5 μM and 4.8 μM, respectively [79], whereas licoagrochalcone A (**70**) was shown to have antiplasmodial activity against chloroquine-sensitive and chloroquin-resistant strains of *Plasmodium falciparum*; the IC_50_ values were determined as 12.7 and 12.0 μM, respectively [81]. Two other chalcones from *Erythrina abyssinica,* homobutein (**19**) (IC_50_ = 15.0 and 16.1 μM, respectively) and 5-prenylated derivative of butein (**16**) (IC_50_ = 10.3 and 11.2 μM, respectively) were also active against both strains for *Plasmodium falciparum*.

**Figure 18:**
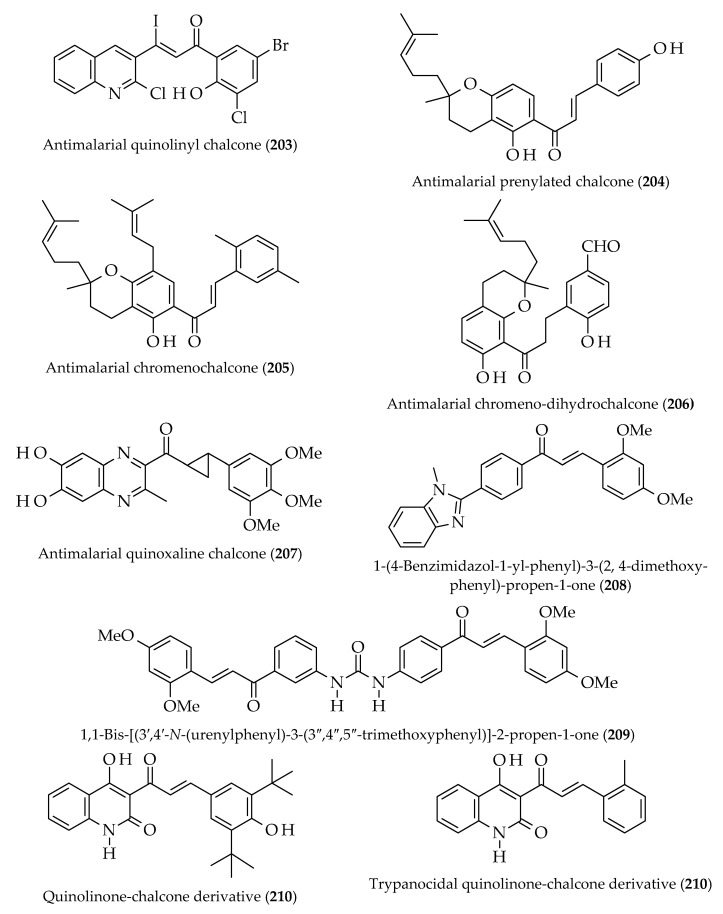
Structures of some synthetic antiparasitic chalcones.

Inspired by the observed antiparasitic activity of naturally occurring chalcones, several synthetic chalcones with potential antiparasitic activity were introduced. Ugwu et al. [119] reported that several synthetic chalcone derivatives of the structural classes chromanochalcones, chromeno-dehydrochalcones, quinolinyl chalcones, morachalcones, prenylated chalcones, chromenochalcones, quinoxaline chalcones, chalcone sulphonamides, and licochalcones could exhibit antimalarial activity, and some of them were even active against chloroquin-resistant *P. falciparaum*. Structures of some of those antimalarial chalcone derivatives (**203**–**207**) are presented in Figure 18. Yadav et al. [120] synthesized 27 antimalarial chalcones, and from the antiplasmodial screening of these compounds, chalcone derivative 1-(4-benzimidazol-1-yl-phenyl)-3-(2, 4-dimethoxy-phenyl)-propen-1-one (**208**) appeared as the most potent one with an IC_50_ value of 1.1 μg/mL against *P. falciparum*. Among the 27 compounds tested, the presence of two methoxyl functionalities at positions 2 and 4 appeared to be optimum for antimalarial activity followed by 3,4-dimethoxy and 2,5-dimethoxy with moderate activity. It was noted that 3,4,5-trimethoxy series of derivatives displayed weak activity, probably because of the stearic hindrance at the binding site of the enzyme. Several dimeric chalcone derivatives were synthesized and their antimalarial potential was evaluated using in vitro globin hydrolysis, β-hematin formation, and murine *Plasmodium berghei* [121]. Among the deimeric chalcone derivatives, 1,1-bis-[(3′,4′-*N*-(urenylphenyl)-3-(3″,4″,5″-trimethoxyphenyl)]-2-propen-1-one (**209**) (Figure 18) was found to be the most active antimalarial candidate.

Quinolinone-chalcone derivatives were synthesized and assessed for their antiparasitic activity against the mammalian stages of *Trypanosoma brucei* and *Leishmania infantum* [122]. Among the compounds, quinolinone-chalcone derivative **210** exhibited the most prominent activity against both parasites, particularly against *Leishmania infantum* (IC_50_ = 1.3 μM). Another derivative (**211**) (Figure 18) showed significant trypanocidal activity (IC_50_ = 2.6 μM). These are just a few examples of antiparasitic synthetic chalcone derivatives, and there are many more examples are available in the literature [123,124].

### 4.7. Immunoregulatory Activity

**Figure 19:**
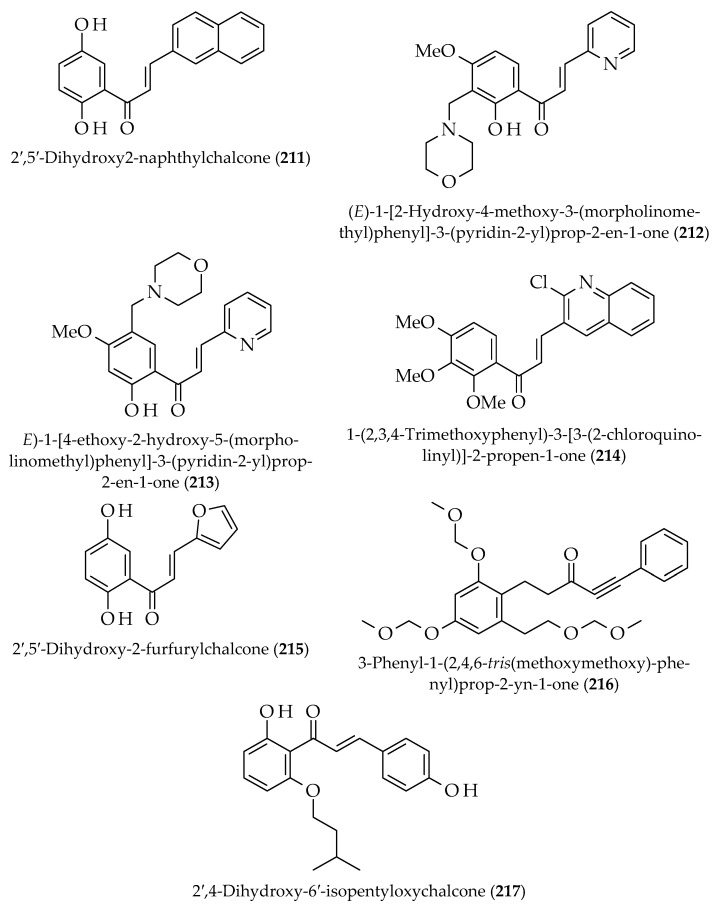
Structures of some synthetic immunomodulatory chalcones.

Natural chalcones mallotophilippens (**74**–**76**) displayed immunoregulatory activity [68]. The immunomodulatory activity of synthetic sulfonamide chalcone derivatives in mice infected with filarial parasites, including Brugia malayi, has recently been studied [125]. Lee et al. [126] reviewed the potential immunomodulatory effects of natural and synthetic chalcones, and several synthetic chalcone derivatives were reported to possess immunomodulatory properties, mediated through multiple mechanisms, e.g., actions on dendritic cells, neutrophils, basophils, innate lymphoid cells, microglial cells, and T cells. Figure 19 shows some examples of synthetic immunomodulatory chalcone derivatives.

Synthetic chalcone analogs, including 2′,5′-dihydroxy2-naphthylchalcone (**211**), could act on neutrophil degranulation and superoxide anion generation, while heterocyclic chalcones, namely (*E*)-1-[2-hydroxy-4-methoxy-3-(morpholinomethyl)phenyl]-3-(pyridin-2-yl)prop-2-en-1-one (**212**) and (*E*)-1-[4-ethoxy-2-hydroxy-5-(morpholinomethyl)phenyl]-3-(pyridin-2-yl)prop-2-en-1-one (**213**) (Figure 19), were found to inhibit the generation of superoxide anion and elastases [126]. Similarly, 1-(2,3,4-trimethoxyphenyl)-3-(3-(2-chloroquinolinyl)-2-propen-1-one (**214**) could suppress the production of elastase and superoxide anion, and LTB4-(leukotriene B4) release in human neutrophils. The *N*-formylmethionyl-leucyl-phenylalanine (fMLP)-stimulated respiratory burst in neutrophils was found to be inhibited by the synthetic chalcone analog, 2′,5′-dihydroxy-2-furfurylchalcone (**215**). An unusual synthetic chalcone derivative, 3-phenyl-1-(2,4,6- tris(methoxymethoxy)phenyl)prop-2-yn-1-one (**216**), was shown to possess immunomodulatory properties, whereas 2′,4-dihydroxy-6′-isopentyloxychalcone (**217**) could modulate an innate immune response [126]. These are just a few examples of synthetic chalcone derivatives that can modulate an immune response, and there are many more similar examples available in the literature [127].

## 5. Conclusions

Over the years, thousands of chalcones and their derivatives were isolated from natural sources, and many of those were screened for potential bioactivities, mainly including anticancer, anti-inflammatory, antimicrobial, antioxidant, and antiparasitic properties. The d of bioactive naturally occurring chalcones has inspired total or partial synthesis of chalcone analogs as well as minor structural modifications of natural chalcones forming a large collection of bioactive synthetic chalcone derivatives, some of which have enhanced bioactivities and/or reduced toxicities compared to relevant natural chalcones. This review article has demonstrated how bioinspired synthesis of chalcone derivatives can potentially introduce a new chemical space for the exploitation for new drug discovery.

## Figures and Tables

**Table 1:** Synthesis of some chalcone and chalcone derivatives [7,8,9].

Approach	Compound 1	Compound 2	Reaction Conditions	Synthesize Chalcone and Its Derivatives
Microwave	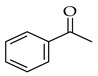	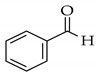	NaOH/MeOH, microwave radiation at 110 W, 55 °C	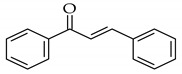 (**1**)
			I_2_-Al_2_O_3_, microwave radiation for 80 s	
Ionic liquids	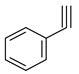	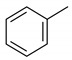	HBr, Bmim(OTs), 12 h, 100 °C	
Claisen-Schmidt	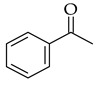	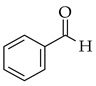	Base	
Using SOCl_2_	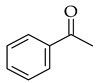	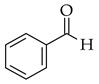	SOCl_2_/EtOH	
Alkynes and aldehydes	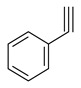	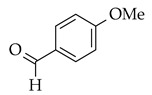	Amberlyst-15, DCM, at r.t.	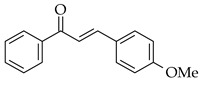 (**2**)
Sonogashira coupling	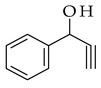	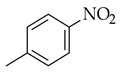	[PdCl_2_(PPh_3_)_2_], Et_3_N, THF, heat	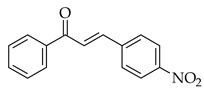 (**3**)
Cross-coupling	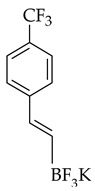	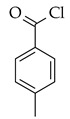	[PdCl_2_(dtbpf)], K_2_CO_3_, 1,4-dioxane, microwave radiation for 30 min at 140 °C	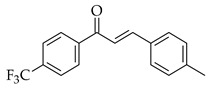 (**4**)
Wittig reaction	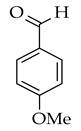	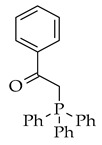	Br-, NaOEt, toluene, microwave radiation for 5 min	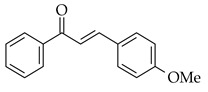 (**2**)
Claisen-Schmidt	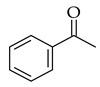	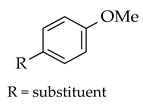	Zeolite ultrasound	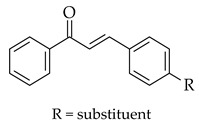 (**5**)
	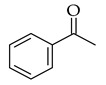	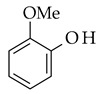	NaOH base	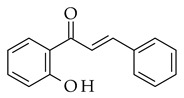 (**6**)
Solvent free	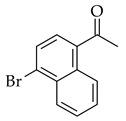	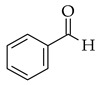	SiO_2_, H_2_SO_4_, 80 °C	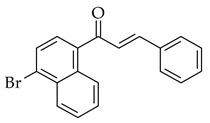 (**7**)
Suzuki reaction	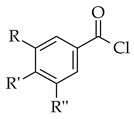	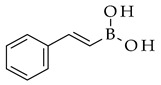	(PPh_3_)_4_Pd^(0)^, Cs_2_CO_3_, toluene	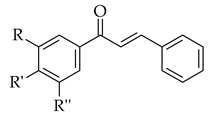 (**8**)
	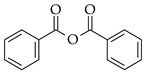	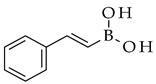	PdCl_2,_ Na_2_CO_3_, water/acetone	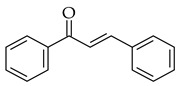 (**1**)
	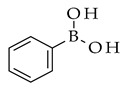	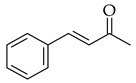	Pd	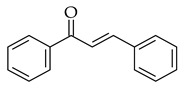 (**1**)
Base-driven	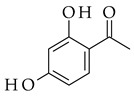	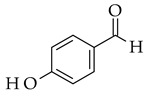	KOH/EtOH, heat, 1–5 h	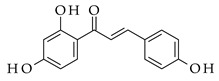 (**9**)
	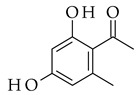	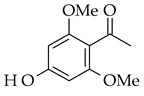	NaOH	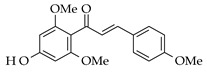 (**10**)

Al_2_O_3_: aluminum oxide; Bmim (OTs): 1-butyl-3-methyl-1*H*-imidazolium 4-methylbenzenesulfonate; DCM: dichloromethane; EtOH: ethanol; Et_3_N: triethylamine; NaOEt: sodium ethoxide; HBr: hydrobromic acid; H_2_SO_4_: sulfuric acid; K_2_CO_3_: potassium carbonate; KOH: potassium hydroxide; MeOH: methanol; Na_2_CO_3_: sodium carbonate; NaOH: sodium hydroxide; Pd: palladium; PdCl_2_: palladium chloride; PdCl_2_(PPh_3_)_2_: bis(triphenylphospine)palladium dichloride; PdCl_2_(dtbpf): 1,1′-bis(di-*tert*-butylphosphino)ferrocene]dichloropalladium; (PPh_3_)_4_Pd^(0)^: tetrakis(triphenylphosphine)palladium(0); SiO_2_: silicon dioxide; SOCl_2_: thionyl chloride; THF: tetrahydrofuran.

**Table 2:** Bioactivities of some naturally occurring chalcones.

Bioactivities		Chalcones	Sources	References
Anti-acetylcholinesterase		Isosalipurposide (**66**) Naringenin chalcone (**78**)	*Acacia cyanophylla*	[50]
Anti-arthritic		Cardamonin (**48**)	*Boesenbergia rotunda*	[51]
Anticancer		Butein (**16**)	*Toxicodendron vernicifluum*	[52]
			*Butea dahlia*	[22,53]
		Calomelanone (**17**)	*Stevia lucida*	
		Deoxydihydroxanthoangelol H (**49**)	*Angelica keiskei*	[54]
		2,6-Dihydroxy-4-methoxychalcone (**52**)		
		Dimethyl-cardamonin (**27**)	*Syzygium campanulatum*	[55]
		Echinantin (**59**)	*Piper methysticum & Alpinia pricei*	[56]
		Flavokawain A (**60**)	*Piper methysticum*	
		Flavokawain B (**28**)		
		Flavokawain B (**28**)	*Alpinia pricei*	[57]
		Flavokawain C (**18**)	*Piper methysticum*	[22,53]
		Homobutein (**19**)	*Butea frondosa*	
		2′-Hydroxy-4′,5′-dimethoxychalcone (**61**)	*Sarcandra hainanensis*	[58]
		4-Hydroxychalcone (**20**)	*Glycyrrhiza glabra*	[22,53]
		4′-Hydroxychalcone (**21**)		
		4-Hydroxyderricin (**62**)	*Angelica keiskei*	[59]
		Isobavachalcone (**63**)		[54]
		Isobavachalcone (**63**)	*Psoralea corylifolia*	[60]
		Isoliquiritigenin (**22**)	*Piper methysticum*	[57]
			*Alpinia pricei*	
			*Glycyrrhiza glabra*	[22,53]
		Isosalipurposide (**66**)	*Helichrysum maracandicum*	[61]
		Lonchocarpin (**41**)	*Pongamia pinnata*	[41]
		Marein (**77**)	*Glycyrrhiza glabra*	[22,53]
		4-Methoxychalcone (**2**)		
		Phloretin (**23**)	*Stevia lucida*	[22,53]
		2,3,4-Trimethoxy-2′-hydroxychalcone (**82**)	*Piper methysticum*	[62]
		Xanthoangelol (**83**)	*Angelica keiskei*	[59]
		Xanthoangelol I (**91**)		[54]
		Xanthoangelol J (**92**)		
Antidiabetic		2′,6′-Dihydroxy-4′-methoxychalcone (**54**)	*Piper claussenianum*	[63]
		4-Hydroxyderricin (**62**)	*Angelica keiskei*	[64]
		Xanthoangelol (**83**)		
Anti-inflammatory		Flavokawain B (**28**)	*Alpinia pricei*	[65]
		Isoliquiritigenin (**22**)		
		Licoagrochalcone A (**70**)	*Glycyrrhiza inflata*	[66]
		Licochalcone B (**68**)		
		Licochalcone C (**71**)	*Glycyrrhiza glabra*	[67]
		Mallotophilippens C (**74**)	*Mallotus philippinensis*	[68]
		Mallotophilippens D (**75**)		
		Mallotophilippens E (**76**)		
Antimicrobial				
	Antibacterial	2′,4′-Dihydroxychalcone (**51**)	*Zuccagnia punctata*	[69]
		3,2′-Dihydroxy-2,4,4′,6′-tetramethoxychalcone (**55**)	*Piper hispidum*	[70]
		2′-Hydroxy-2,4,4′,6′-tetramethoxychalcone (**56**)		
		2′-Hydroxy-2,3,4,4′,6′-pentamethoxychalcone (**57**)		
		Isoliquiritigenin (**22**)	*Apis mellifera*	[71]
	Antifungal	1-(5,7-Dihydroxy-2,2,6-trimethyl-2H-1-benzopyran-8-yl)-3-phenyl-2-propen-1-one [58]	*Mallotus philippinensis*	[72]
		3,2′-Dihydroxy-2,4,4′,6′-tetramethoxychalcone (**55**)	*Piper hispidum*	[70]
		2′-Hydroxy-2,4,4′,6′-tetramethoxychalcone (**56**)		
		2′-Hydroxy-2,3,4,4′,6′-pentamethoxychalcone (**57**)		
		4′-Hydroxyrottlerin (**64**)	*Mallotus philippinensis*	[72]
		Isoliquiritigenin (**22**)	*Apis mellifera*	[71]
		Kamalachalcone E (**67**)	*Mallotus philippinensis*	[72]
		Rottlerin (**65**)		
		2′,3,4,4′-Tetrahydroxy-3′-geranylchalcone (**85**)	*Artocarpus nobilis*	[73]
		2′,3,4,4′-Tetrahydroxy-3′-(6-hydroxy-3,7-dimethyl-7-octadienyl)chalcone (**79**)		
		2′,4′,4-Trihydroxy-3′-geranylchalcone (**83**) [xanthoangelol]		
		2′,4′,4-Trihydroxy-3′-(6-hydroxy-3,7-dimethyl-7-octadienyl)chalcone (**80**)		
		2′,4′,4-Trihydroxy-3′-[2-hydroxy-7-methyl-3-methylene-6-octenyl-chalcone] (**81**)		
	Antiviral	Dihydrochalcone diglycoside (**50**)	*Thalassodendrin ciliatum*	[74]
		Echinantin (**59**)	*Glycyrrhiza inflata*	[75]
		Isoliquiritigenin (**22**)		
		Licochalcone A (**40**)	*Quercus coccifera*	[76]
			*Glycyrrhiza inflata*	[75]
		Licochalcone B (**68**)	*Quercus coccifera*	[76]
		Licochalcone D (**72**)	*Glycyrrhiza inflata* *Quercus coccifera*	[76]
		Licochalcone G (**73**)		
		Tetrahydroxy-methoxychalcone (**69**)		[76]
		Xanthoangelol (**83**)	*Angelica keiskei*	[77]
		Xanthoangelol F (**84**)		
		Xanthoangelol D (**89**)		
		Xanthoangelol E (**90**)		
		Xanthoangelol B (**86**)		
		Xanthoangelol G (**87**)		
		Xanthodeistal A (**88**)		
Antioxidant		Licochalcone C (**71**)	*Glycyrrhiza glabra*	[67]
		Isosalipurposide (**66**) Naringenin chalcone (**78**)	*Acacia cyanophylla*	[50]
Antiparasitic		(*E*)-1-(2,4-Dihydroxy-3-(3-methylbut-2-en-1-yl)phenyl)-3-phenylprop-2-en-1-one (**53**)	*Lonchocarpus* sp.	[78]
		Flavokawain B (**28**)	*Polygonum ferrugineum*	[79]
		Licochalcone C (**71**)	*Glycyrrhiza glabra*	[80]
		Licoagrochalcone A (**70**)	*Erythrina abyssinica*	[81]
Immunoregulatory		Mallotophilippens C (**74**)	*Mallotus philippinensis*	[68]
		Mallotophilippens D (**75**)		
		Mallotophilippens E (**76**)		

## Data Availability

All relevant data were presented as an integral part of this manuscript.
